# Evaluating Radiological Awareness of Carbon Monoxide Poisoning Among Physicians in Saudi Arabia

**DOI:** 10.7759/cureus.97081

**Published:** 2025-11-17

**Authors:** Saba Aldusaymani, Abdulaziz Zaid Alhayli, Weaam I Faraj, Hatim S Sendy, Adel Abdullah Alobaidi

**Affiliations:** 1 Radiology, King Saud Medical City, Riyadh, SAU; 2 Diagnostic Radiology, Aseer Central Hospital, Aseer, SAU; 3 Forensic Medicine, Jeddah Forensic Medicine Center, Jeddah, SAU; 4 General Practice, King Salman General Hospital, Riyadh, SAU; 5 Vascular and Interventional Radiology, Diriyah Hospital, Riyadh, SAU

**Keywords:** awareness, carbon monoxide, knowledge, physicians, poisoning

## Abstract

Background: A poisonous, colorless, odorless, and tasteless gas is carbon monoxide (CO). High-level exposure to CO can result in serious illness or death, and CO poisoning is now recognized as a critical public health concern worldwide. The purpose of this study was to evaluate Saudi Arabian physicians' radiological knowledge of CO intoxication.

Methods: This online survey research, which was cross-sectional, assessed doctors' knowledge and awareness of CO poisoning in Saudi Arabia. Licensed physicians were invited to complete an online questionnaire covering demographics and work data, exposure to CO poisoning cases, training history, radiological awareness, and physicians' practice and attitude towards CO poisoning. The questionnaire was validated for clarity, reliability, and relevance and was shared on social media platforms for participation.

Results: Among 393 eligible physicians, 127 (32.3%) had received training on CO poisoning and its radiological manifestations, while 137 (34.9%) had encountered suspected CO poisoning cases in their clinical practice. Overall, 340 (86.5%) physicians demonstrated poor knowledge and awareness of radiological indicators of CO poisoning, while only 53 (13.5%) exhibited adequate awareness. Higher awareness was significantly associated with greater years of experience (p<0.05).

Conclusions: This study reveals that most physicians in Saudi Arabia lack sufficient knowledge of CO poisoning and its radiological indicators. Although about one-third had relevant exposure and training, the perceived training effectiveness was low. Senior physicians displayed greater awareness, and most respondents acknowledged the importance of identifying radiological signs.

## Introduction

When carbon-containing substances burn incompletely, carbon monoxide (CO), a colorless, odorless, and tasteless gas, is created. Common sources include charcoal grills, gas generators, and wood-burning stoves [1]. CO poisoning is preventable yet remains a potentially fatal condition that affects people of all ages and demographics. Acute exposure can result in a variety of symptoms, including seizures, migraines, flu-like symptoms, cognitive impairments, coma, and even death. Up to 90% of CO poisoning cases present with headaches as a primary symptom [2-6].

Every year in the United States, CO poisoning causes over 50,000 ER visits and 500 fatalities, making it a serious worldwide public health issue. Additionally, it is a major global contributor to poisoning-related morbidity and mortality, with low- and middle-income countries accounting for 43% of all CO-related deaths [4,7,8]. The actual incidence of CO poisoning is likely much higher due to underreporting and misdiagnoses. It is now estimated that there are 137 instances of CO poisoning for every million people globally. Over the previous 25 years, the death rate has decreased, although the global incidence has been constant; the annual mortality is currently estimated at 4.6 deaths per million population [9,10].

In Saudi Arabia, CO ranked as the second most common poisoning agent among poisonings received at Riyadh's King Abdulaziz Medical City, a tertiary care institution that opened in 2016 through 2020. Globally, the most common circumstances for exposure to CO are related to the use of stoves and gas-heating appliances, particularly in winter [11,12]. Meanwhile, the circumstances leading to CO poisoning may slightly differ in Saudi Arabia. The Saudi climate is warm or hot in most seasons over a wide range of the kingdom, reducing the use of heating appliances. Aldossary et al. [13] in Dammam, Saudi Arabia, found that 64% of CO fatalities were caused by fire accidents. Similarly, Al-Asmari et al. [14] revealed that fire incidents were the primary cause of CO poisonings and CO-related deaths in the Mekkah area of Saudi Arabia.

Diagnosing CO poisoning in emergency settings can be challenging, necessitating a thorough differential diagnosis. Patients with suspected CO exposure often exhibit elevated carboxyhemoglobin levels [15].

CO poisoning can produce both focal and generalized neurological symptoms. When evaluating brain injury, magnetic resonance imaging (MRI) is favored over computed tomography (CT) [16-18]. Despite the significant health risks associated with CO exposure, many healthcare professionals may be unaware of CO poisoning's radiological findings. Radiological imaging, including chest X-rays, CT, and MRI, plays a critical role in diagnosing and monitoring CO poisoning cases [19,20]. Lack of adequate training could lead healthcare providers to miss crucial radiological clues. Consequently, diagnosis and therapy are delayed.

The purpose of this study is to assess the radiological awareness of CO poisoning among physicians in Saudi Arabia and evaluate its clinical impact. Using a cross-sectional study design with a stratified random sampling approach to ensure a representative sample of physicians from various specialties and regions.

## Materials and methods

The Biomedical Research Ethics Committee of the Al Qunfudah Faculty of Medicine at Umm Al-Qura University in Saudi Arabia gave its clearance for this work (ID: HAPO-02-K-012-2023-05-1633, Date: 22/5/2023). Respondents gave consent before participation, and they were assured of data confidentiality and informed of the public health importance of the study.

A cross-sectional, web-based survey research was carried out to evaluate Saudi Arabian physicians' awareness and understanding of CO poisoning. The study included all licensed physicians practicing in the country. Medical students and incomplete responses were excluded.

Literature research and expert consultation served as the foundation for the development of an online questionnaire. The questionnaire received a Cronbach's alpha of 0.72 after being examined and verified by three separate experts for appropriateness, clarity, and reliability. The anonymous questionnaire was distributed through social media platforms over a period of four months.

The questionnaire consisted of four sections: (1) demographic and professional data (age, gender, specialty, place of work, and years of experience); (2) exposure to cases of CO poisoning and training history; (3) radiological awareness of CO poisoning; and (4) physicians' practices and attitudes towards radiological awareness of CO poisoning and its importance.

Statistical Product and Service Solutions (SPSS, version 26; IBM SPSS Statistics for Windows, Armonk, NY) was used to analyze the data. The aggregate of the right answers was used to determine the overall knowledge score; each right response was worth one point; if not, it was worth zero. The scores were normalized as percentages and varied from 1 to 24. Individuals who received a score below 60% were categorized as having inadequate knowledge, while those scoring 60% or more were classified as having good knowledge. Descriptive analyses for categorical data were presented in tables as frequencies and percentages. Cross-tabulations were used to examine factors associated with radiological awareness and its influence on practice and perceptions. The exact probability test for tiny distributions and Pearson's chi-squared test were used to assess statistical significance. A p-value of less than 0.05 was considered to be significant.

## Results

The survey was filled out by 393 eligible physicians, of whom 109 (27.7%) were from the central region, 89 (22.6%) were from the western region, and 86 (21.9%) were from the eastern region. Other respondents were from the other regions. The mean age of physicians was 26.1 ± 11.8 years, with a range of 21-60 years. Females comprised 222 (56.5%) of respondents. Regarding specialties, 108 (27.5%) were general practitioners, 27 (6.9%) radiologists, and 19 (4.8%) emergency physicians, while the remainder (60.8%) were from other specialties. Respondents included residents (158, 40.2%), service residents (110, 28%), and consultants (64, 16.3%). Most physicians (237, 60.3%) had 1-5 years of experience, 70 (17.8%) had 6-10 years, and 35 (8.9%) had more than 10 years. A majority (355, 90.3%) worked in governmental hospitals. Of the participants, 127 (32.3%) had received training on CO poisoning and its radiological findings, while 137 (34.9%) had encountered suspected CO poisoning cases in clinical practice (Table 1).

**Table 1:** Socio-demographic and work data of study physicians in Saudi Arabia (N=393)

Socio-demographic data	n	%
Region		
Central Region	109	27.7%
Western Region	89	22.6%
Eastern Region	86	21.9%
Northern Region	55	14.0%
Southern Region	54	13.7%
Age in years		
21-30	228	58.0%
31-40	95	24.2%
41-50	44	11.2%
> 50	26	6.6%
Gender		
Male	222	56.5%
Female	171	43.5%
Specialty		
General practitioner	108	27.5%
Radiology	27	6.9%
Emergency medicine	19	4.8%
Others	239	60.8%
Specialty position		
Intern	7	1.8%
Service resident	110	28.0%
Resident	158	40.2%
Specialist	54	13.7%
Consultant	64	16.3%
Experience (years in practice)		
1-5 years	237	60.3%
6-10 years	70	17.8%
10-20 years	51	13.0%
> 20 years	35	8.9%
Place of practice		
Governmental	355	90.3%
Private	38	9.7%
Have you previously undergone training on carbon monoxide poisoning and its radiological manifestations?		
Yes	127	32.3%
No	266	67.7%
Have you encountered patients with suspected carbon monoxide poisoning in your clinical practice?		
Yes	137	34.9%
No	256	65.1%

Table 2 shows that a total of 43.5% of participants reported awareness of CO poisoning's radiological findings. Commonly recognized findings included consolidation (36.9%) and ground-glass opacities (36.1%). Only 48.9% acknowledged that imaging studies could aid in diagnosing CO poisoning, and 37.9% identified hypodense areas in the white matter on CT scans as indicative of brain damage caused by CO poisoning.

**Table 2:** Radiological awareness of carbon monoxide poisoning among physicians in Saudi Arabia

Awareness items		n	%
Are you aware of the radiological findings associated with carbon monoxide poisoning?	Yes	171	43.5%
	No	222	56.5%
Radiological findings are commonly associated with carbon monoxide poisoning	Ground glass opacities	142	36.1%
	Consolidation	145	36.9%
	Air bronchograms	134	34.1%
	Tree-in-bud appearance	84	21.4%
	None of the above	103	26.2%
How can imaging studies be used to diagnose carbon monoxide poisoning?	By showing characteristic radiological abnormalities	192	48.9%
	By detecting the presence of carbon monoxide in the bloodstream	111	28.2%
	By measuring the oxygen saturation in the blood	80	20.4%
	By assessing the size of the heart	10	2.5%
What are the common radiological findings associated with brain damage caused by carbon monoxide poisoning on computed tomography scans?	Hypodense areas in the white matter	149	37.9%
	Hemorrhagic lesions	60	15.3%
	Increased gray matter density	83	21.1%
	Cerebral edema	101	25.7%
What are the most commonly affected regions of the brain in carbon monoxide poisoning, and how are they affected?	Frontal cortex, causing personality changes	41	10.4%
	Hippocampus, causing memory impairment	99	25.2%
	Basal ganglia, causing movement disorders	174	44.3%
	Cerebellum, causing balance problems	79	20.1%
Which imaging modality is most commonly used for the diagnosis of carbon monoxide poisoning?	Chest X-ray	139	35.4%
	Computed tomography scan	134	34.1%
	Magnetic resonance imaging	75	19.1%
	Ultrasound	14	3.6%
	None of the above	31	7.9%
Can you distinguish carbon monoxide poisoning radiological findings from those of other pulmonary diseases?	Yes	173	44.0%
	No	220	56.0%
What is the treatment for carbon monoxide poisoning?	Hyperbaric oxygen therapy	113	28.8%
	Oxygen therapy	152	38.7%
	Antidote therapy	34	8.7%
	None of the above	12	3.1%
	Not sure	82	20.9%
What is the role of radiological imaging in the management of carbon monoxide poisoning?	To diagnose carbon monoxide poisoning	62	15.8%
	To monitor the severity of carbon monoxide poisoning	67	17.0%
	To assess the response to treatment	42	10.7%
	All of the above	191	48.6%
	None of the above	31	7.9%
Which of the following statements correctly describes the radiological appearance of carbon monoxide poisoning compared to other pathologies?	Carbon monoxide poisoning may show non-specific changes similar to other pathologies	96	24.4%
	Carbon monoxide poisoning typically shows no abnormal radiological findings	92	23.4%
	Carbon monoxide poisoning shows diffuse bilateral hyperdensity in the basal ganglia on computed tomography scans	138	35.1%
	Carbon monoxide poisoning shows a characteristic "snowstorm" appearance on computed tomography scans of the brain	67	17.0%
How can the radiological appearance of carbon monoxide poisoning be used in treatment?	Radiological appearance can be used to predict the likelihood of complications	120	30.5%
	Radiological appearance has no role in the treatment of carbon monoxide poisoning	80	20.4%
	Radiological appearance can be used to determine the need for oxygen therapy	95	24.2%
	Radiological appearance can be used to guide hyperbaric oxygen therapy	98	24.9%
Which of the following is an indicator of successful carbon monoxide poisoning treatment on radiological imaging?	Complete resolution of radiological abnormalities	182	46.3%
	Reduction in the size of the basal ganglia	101	25.7%
	Increase in the number of white matter lesions.	68	17.3%
	Persistence of radiological abnormalities despite treatment	42	10.7%
Which of the following complications of carbon monoxide poisoning can be detected in imaging studies?	Acute respiratory distress syndrome	188	47.8%
	Cardiac arrest	86	21.9%
	Seizures	91	23.2%
	Rhabdomyolysis	28	7.1%

Overall, 340 (86.5%) physicians had poor radiological awareness, while only 53 (13.5%) demonstrated good awareness.

Among respondents, 112 (28.5%) had interpreted imaging from suspected CO poisoning cases, with 58 (51.8%) identifying radiological findings consistent with CO poisoning. Regarding training, 119 (30.3%) received formal training on CO poisoning's radiological findings, but 53 (44.6%) rated it poorly. Regarding perceptions, 318 (80.9%) believed limited clinical exposure affected their knowledge, and 283 (72%) cited limited radiological imaging availability as a barrier to effective diagnosis (Table 3).

**Table 3:** Perception and practice of study physicians regarding carbon monoxide poisoning in Saudi Arabia

Perception and practice	n	%
Have you ever interpreted a chest radiograph or computed tomography scan of a patient with suspected carbon monoxide poisoning?		
Yes	112	28.5%
No	281	71.5%
If yes, were you able to identify radiological findings consistent with carbon monoxide poisoning?		
Yes	58	51.8%
No	18	16.1%
Not sure	36	32.1%
Have you received any formal education or training on carbon monoxide poisoning and its radiological findings?		
Yes	119	30.3%
No	274	69.7%
If yes, please rate the effectiveness of the training program on a scale of 1 to 5, with 1 being not effective at all and 5 being very effective.		
1	24	20.2%
2	29	24.4%
3	36	30.3%
4	15	12.6%
5	15	12.6%
Have you ever encountered a patient with carbon monoxide poisoning who had normal chest radiographs or computed tomography scans?		
Yes	91	23.2%
No	141	35.9%
Not sure	161	41.0%
Do you think that the lack of clinical exposure to carbon monoxide poisoning cases affects your knowledge of the radiological findings associated with carbon monoxide poisoning?		
Yes	318	80.9%
No	75	19.1%
Do you think that the limited availability of radiological imaging in some hospitals and clinics in Saudi Arabia affects the diagnosis and management of carbon monoxide poisoning?		
Yes	283	72.0%
No	110	28.0%
Based on your experience and knowledge, how important do you think it is for physicians to be able to identify radiological findings associated with carbon monoxide poisoning?		
Very important	247	62.8%
Somewhat important	139	35.4%
Not important	7	1.8%

Good awareness was significantly associated with age (41-50 years: 22.7% vs. 21-30 years: 12.3%; p=0.046) and experience (10-20 years: 25.5% vs. 6-10 years: 5.7%; p=0.019). Additionally, 25.5% of physicians with 10-20 years of experience had a significantly higher level of overall awareness compared to 5.7% of others with 6-10 years of experience (p=0.019) (Table 4).

**Table 4:** Factors associated with radiological awareness of carbon monoxide poisoning among physicians in Saudi Arabia P: Pearson X^2^ test; ^: Exact probability test; *: p < 0.05 (significant)

Factors	Overall awareness level				p-value
	Poor		Good		
	n	%	n	%	
Region					0.808
Eastern Region	76	88.4%	10	11.6%	
Northern Region	48	87.3%	7	12.7%	
Southern Region	46	85.2%	8	14.8%	
The Central Region	91	83.5%	18	16.5%	
Western Region	79	88.8%	10	11.2%	
Age in years					0.046*
21-30	200	87.7%	28	12.3%	
31-40	85	89.5%	10	10.5%	
41-50	34	77.3%	10	22.7%	
> 50	21	80.8%	5	19.2%	
Gender					0.985
Male	192	86.5%	30	13.5%	
Female	148	86.5%	23	13.5%	
Specialty					0.410^
GP	97	89.8%	11	10.2%	
Radiology	21	77.8%	6	22.2%	
Emergency medicine	16	84.2%	3	15.8%	
Others	206	86.2%	33	13.8%	
Specialty position					0.557^
Intern	5	71.4%	2	28.6%	
Service resident	96	87.3%	14	12.7%	
Resident	137	86.7%	21	13.3%	
Specialist	49	90.7%	5	9.3%	
Consultant	53	82.8%	11	17.2%	
Experience years in practice					0.019*
1-5 years	206	86.9%	31	13.1%	
6-10 years	66	94.3%	4	5.7%	
10-20 years	38	74.5%	13	25.5%	
> 20 years	30	85.7%	5	14.3%	
Place of practice					0.662
Governmental	308	86.8%	47	13.2%	
Private	32	84.2%	6	15.8%	
Have you previously undergone training on carbon monoxide poisoning and its radiological manifestations?					0.554
Yes	108	85.0%	19	15.0%	
No	232	87.2%	34	12.8%	
Have you encountered patients with suspected carbon monoxide poisoning in your clinical practice?					0.434
Yes	116	84.7%	21	15.3%	
No	224	87.5%	32	12.5%	

Table 5 shows that physicians with good awareness were more likely to interpret imaging correctly (39.6% vs. 26.8%; p=0.046) and to consider identifying radiological findings critical (71.7% vs. 61.5%; p=0.049).

**Table 5:** Effect of physician's awareness level of radiological findings on carbon monoxide poisoning on physicians' practice and perception P: Pearson X^2^ test; ^: Exact probability test; *: p < 0.05 (significant)

Practice & perception	Overall awareness level				p-value
	Poor		Good		
	n	%	n	%	
Have you ever interpreted a chest radiograph or computed tomography scan of a patient with suspected carbon monoxide poisoning?					0.046*
Yes	91	26.8%	21	39.6%	
No	249	73.2%	32	60.4%	
If yes, were you able to identify radiological findings consistent with carbon monoxide poisoning?					0.652
Yes	46	50.5%	12	57.1%	
No	16	17.6%	2	9.5%	
Not sure	29	31.9%	7	33.3%	
Have you received any formal education or training on carbon monoxide poisoning and its radiological findings?					0.530
Yes	101	29.7%	18	34.0%	
No	239	70.3%	35	66.0%	
Have you ever encountered a patient with carbon monoxide poisoning who had normal chest radiographs or CT scans?					0.116
Yes	79	23.2%	12	22.6%	
No	128	37.6%	13	24.5%	
Not sure	133	39.1%	28	52.8%	
Do you think that the lack of clinical exposure to carbon monoxide poisoning cases affects your knowledge of the radiological findings associated with carbon monoxide poisoning?					0.122
Yes	271	79.7%	47	88.7%	
No	69	20.3%	6	11.3%	
Do you think that the limited availability of radiological imaging in some hospitals and clinics in Saudi Arabia affects the diagnosis and management of carbon monoxide poisoning?					0.476
Yes	247	72.6%	36	67.9%	
No	93	27.4%	17	32.1%	
Based on your experience and knowledge, how important do you think it is for physicians to be able to identify radiological findings associated with carbon monoxide poisoning?					0.049*^
Very important	209	61.5%	38	71.7%	
Somewhat important	126	37.1%	13	24.5%	
Not important	5	1.5%	2	3.8%	

## Discussion

There is a paucity of literature on the frequency of correct and incorrect diagnoses of CO poisoning in emergency departments. A previous study suggested that misdiagnosis of CO poisoning may occur in up to 98% of cases [21]. The literature reports that misdiagnosis can occur in some instances of CO poisoning due to an ambiguous history, leading to the development of delayed neurological sequelae in such patients [22]. The purpose of the current study was to evaluate Saudi Arabian physicians' radiological knowledge of CO toxicity. The results revealed that most (over three-quarters) of the participating physicians demonstrated poor knowledge and awareness regarding CO poisoning and its radiological assessment. Less than half reported familiarity with the radiological findings of CO poisoning, with consolidation and ground-glass opacities being the most frequently identified features. Almost half were aware that imaging studies can be used to diagnose CO poisoning by showing characteristic radiological abnormalities, but less than half could distinguish the radiological findings of CO poisoning from those of other lung diseases. Approximately one-third of physicians recognized hypodense areas in the white matter as a common radiological finding indicative of brain damage caused by CO poisoning on CT scans. Additionally, fewer than half were aware that complete resolution of radiological abnormalities signifies successful treatment or that imaging findings could predict complications. Acute respiratory distress syndrome was correctly identified as a CO poisoning complication by about half of the respondents.

Older and more experienced physicians exhibited significantly better awareness levels. Surprisingly, radiologists and emergency physicians did not demonstrate significantly greater awareness compared to other specialties, despite being the first responders in CO poisoning cases. This finding highlights the urgent and intense need for launching and implementing awareness-raising programs that principally target the physicians involved in the management of poisoning patients, such as emergency physicians and radiologists. In addition, further studies are required in various medical centers that manage poisoning cases in order to assess the levels of awareness and knowledge regarding the diagnosis and treatment of CO poisoning. This recommendation stems from the lack of published studies that addressed this topic, even though CO is the second most frequent poisoning agent in Saudi Arabia [10].

Differences in medical education and training likely contribute to the defective knowledge levels elicited in the current study. Previous survey studies in Saudi Arabia reported a moderate level. However, medical students' and interns' expertise in risk assessment and treating severely poisoned patients is inadequate [23,24].

Some medical schools have incorporated clinical toxicology into their undergraduate programs. Some countries, such as the United States, have included toxicology training in the curriculum of emergency medical programs [25,26]. However, some Saudi medical colleges have not enlisted clinical toxicology among the courses for undergraduate medical students, and any clinical training is delayed until the internship [24]. The incorporation of clinical toxicology within the curricula of undergraduate medical students, as well as internships and the training of emergency physicians, is essential to increase the efficiency of managing acutely poisoned patients and reducing the burden of delayed poison-related complications.

Limitations 

The use of social media channels for distribution and an online poll are among the study's limitations, which may have introduced selection bias, favoring participation by more technologically engaged physicians and potentially underrepresenting others. In addition, the cross-sectional design captures awareness at a single point in time and does not allow for assessment of causality or change over time. Moreover, the reliance on self-reported data may be subject to response bias or overestimation of knowledge levels.

## Conclusions

This study highlights a critical gap in physicians' knowledge and awareness of CO poisoning in Saudi Arabia, particularly regarding diagnostic modalities and radiologic findings. Although one-third of the participants had encountered cases of CO poisoning or had received relevant training, most rated the effectiveness of the training as low. Older, more experienced physicians had higher levels of awareness. Encouragingly, most respondents acknowledged the importance of identifying radiological findings associated with CO poisoning. To address this gap, it is recommended that CO poisoning and other life-threatening emergencies be given greater emphasis in undergraduate medical curricula. Regular health education sessions for healthcare providers who are frequently exposed to such cases should be implemented. Furthermore, more study is required to determine the reasons behind Saudi physicians' lack of knowledge and to create teaching programs targeted at enhancing radiologists' and clinicians' proficiency in reading imaging results linked to CO poisoning.
